# A Neural Seat for Math?

**DOI:** 10.1371/journal.pbio.0040149

**Published:** 2006-04-11

**Authors:** Liza Gross

Abstract mathematical reasoning is often treated as a uniquely human endeavor. But many species, from pigeons to primates, show some ability to grasp the concept of number, suggesting that these numerical abilities represent the evolutionary building blocks of higher math in humans. Sophisticated symbolic number processing in adults recruits a region of the brain called the intraparietal sulcus (IPS). While children can grasp basic math concepts relating to size and number before they know the words that describe them, very little is known about the neural basis of these abilities. Does the IPS support the relatively simple numerical tasks of childhood as well as the sophisticated numerical calculations that adults learn to perform?

Behavioral studies show that adults respond similarly to nonsymbolic numerical stimuli (arrays of dots) and symbolic numerical stimuli (Arabic numerals), suggesting that a common pathway supports both tasks. But neuroimaging studies have not resolved whether the same brain pathway is involved in both symbolic and nonsymbolic number processing. In a new study, Jessica Cantlon, Elizabeth Brannon, Elizabeth Carter, and Kevin Pelphrey at Duke University used functional magnetic resonance imaging (fMRI) to investigate how the IPS responds to nonsymbolic numerical values in adults and preschool-aged children. They show that the brain circuitry governing nonsymbolic number processing is already in place very early in human development.

In the study, adults and four-year-olds lay in a scanner while passively viewing a continuous stream of visual arrays on a computer screen. The arrays were designed to elicit differences in brain response to stimuli that were either novel in number or novel in shape. This study design operates under the assumption that neurons tuned to a particular stimuli (numbers, for example) will stop responding when exposed to a standard stimulus (16 circles) over and over, but will respond to stimuli that deviate from the norm (six or 32 circles). Every so often, a deviant number or shape (a triangle or square in place of a circle) was mixed in with the standard stimuli. Participants pressed a button when a crossbar in the center of the visual display turned red to maintain focus. Cantlon et al. analyzed the fMRI data to determine which brain regions responded to both types of deviant stimuli in the adults and children.

Number deviants produced a much greater bilateral response in the IPS of adults compared with shape deviants, with activity extending into the inferior and superior parietal lobules (SPL). This response was confirmed by an alternate measure of brain activation based on blood oxygen level, which rose significantly three to 7.5 seconds after the number of elements changed. Brain regions that responded to shape deviants were concentrated in the ventral temporal-occipital cortex.

fMRI results for the children showed that number deviants produced a significant response in and around the right IPS and the right SPL. Brain response to shape deviants was similar to that observed in adults. The location and pattern of brain activity in the preschoolers resembled that reported in studies of nonsymbolic numerical processing and basic math ability in adults. By four years old, children's brains already selectively respond to nonsymbolic numerical values, suggesting that the neural networks for number processing are established early in life.

How to explain the finding that IPS activity was bilateral in adults and concentrated in the right hemisphere in the four-year-olds? It could be that the left hemisphere acquires more sophisticated math-related functions over time while the right remains relatively stable. But since some children showed more activity in the left IPS, the researchers warn that future study will have to determine whether this pattern is unique to kids.

Overall, these results indicate that the brain dedicates a region to cultivating numerical abilities early in development. The IPS provides the neurobiological platform for nonsymbolic numerical processing in young children, then supports the expanding capacity for higher-math operations in adulthood. Six-month-olds also have an abstract numerical sense, suggesting that the IPS may even underlie numerical processing in infancy. Much remains to be learned about how children learn to count and match words with symbolic representations of numbers, but these results suggest that focusing on the IPS might help relate biology to behavior to answer some of these questions, and perhaps shed light on the evolution of numerical cognition.

## 

**Figure:**
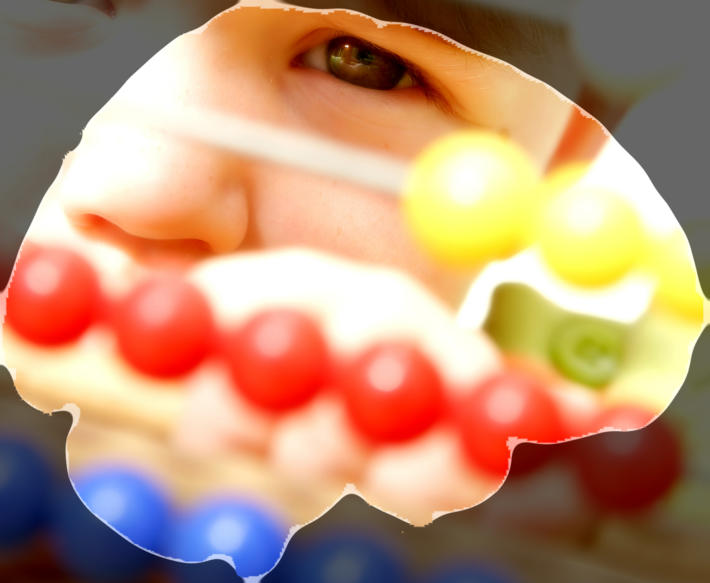
The brain circuits for comprehending math are already in place early in development.

